# Supplementary material for: The immediate-early protein 1 of human herpesvirus 6B interacts with NBS1 and inhibits ATM signaling
Source: EMBO Rep. 2024 Jan 2;25(2):725–44. doi: 10.1038/s44319-023-00035-z (PMC10897193; doi:10.1038/s44319-023-00035-z)
Supplement: Supplementary file 1 — Appendix [file 44319_2023_35_MOESM1_ESM.pdf]

APPENDIX for

The immediate-early protein 1 of human herpesvirus 6B interacts with NBS1 and inhibits ATM signaling

Table of Contents

Appendix Figure S1. Characterization of MOLT-3 infected cells and U2OS clone C10 and C102 expressing IE1, related to Fig. 1 2

Appendix Figure S2. Representative images of the phenotypes quantified in MOLT-3 and U2OS cells and validation of transient expression of IE1 in U2OS cells, related to Fig. 2 4

Appendix Figure S3. Characterization of additional DNA repair reporter assays and associated controls in cells expressing IE1, related to Fig. 3 5

Appendix Figure S4. Representative images and analyses of the colocalization of IE1 with NBS1 in U2OS cells, related to Fig. 4 7

Appendix Figure S5. Representative image of the quantification presented in Fig. 5 and validation of Flag-IE1 expression in U2OS cells 8

Appendix Figure S6. Validation of the expression and cellular localization of IE1 and NBS1 fragments used in this study, related to Fig. 6 9

Appendix Figure S7. Validation of the expression and cellular localization of IE1 fragments used in to study the inhibition of ATM by IE1, related to Fig. 7 13

Appendix Figure S8. Validation of NBS1 and ATM knockdown and its impact on cell viability 16

Appendix table S1. Plasmids used in this study 17

Appendix table S2. Primers, siRNA, shRNAs, and FISH probe sequences used in this study 18

Appendix table S3. Antibodies used in this study 19

Appendix References 20

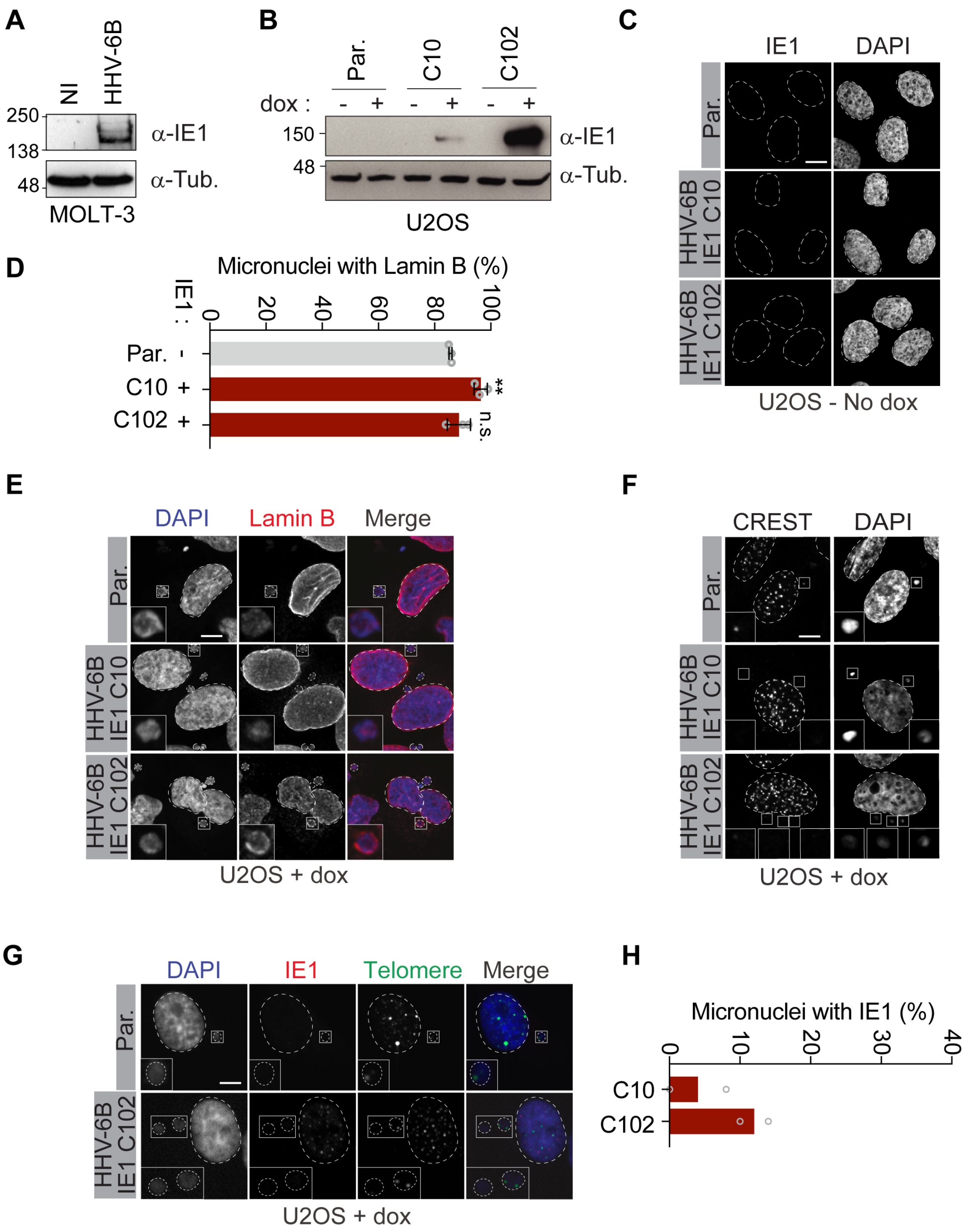

Appendix Figure S1. Characterization of MOLT-3 infected cells and U2OS clone C10 and C102 expressing IE1, related to Fig. 1.

(A-B) Whole cell extracts (WCEs) from infected MOLT-3 (A) and U2OS cells treated with or without 1 µg/mL Dox (B) were immunoblotted for IE1. α-tubulin (Tub.) was used as a loading control. NI: non-infected, Par.: parental

cell line. (C, F, G) Representative images of parental (Par.) U2OS cells and IE1-expressing U2OS stable cell lines corresponding to Fig. 1B, D, and E, respectively. Cells were treated as described in Fig. 1B and either processed for IE1 (C (without DOX), G) or centromeres (CREST) immunofluorescence (F) or FISH for telomere detection (G). (D-E) Dox-inducible U2OS (Par.) and U2OS IE1 stable cell lines were treated as in Fig. 1B and immunostained for lamin B. Quantification of lamin B-positive micronuclei is presented in (D) (n = 3 biological replicates) and representative images are in (E). (H) Quantification of micronuclei colocalizing with IE1 foci. IE1-expressing cells were treated as in Fig. 1B, fixed, and immunostained for IE1 (n = 2 biological replicates, > 100 nuclei/condition).

Data information: In (D), data are presented as mean ± SD. n.s. not significant, **$p \leq 0.01$, (One-way ANOVA with Dunnett's multiple comparison test by comparing to parental cell line (Par.)). In (H), data are presented as mean ± SD. Scale bars = 5 µm.

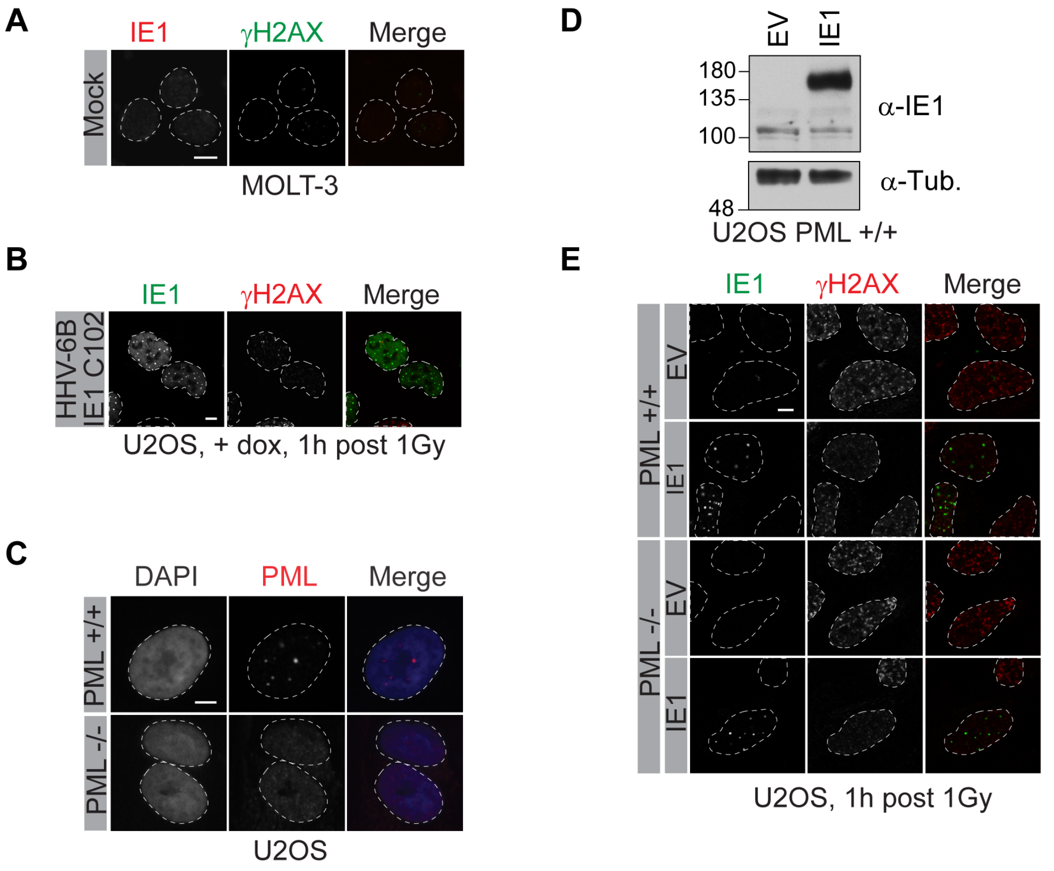

Appendix Figure S2. Representative images of the phenotypes quantified in MOLT-3 and U2OS cells and validation of transient expression of IE1 in U2OS cells, related to Fig. 2.

(A) Representative images of untreated MOLT-3 cells immunostained for γH2AX. Cells were treated as in Fig 2A. (B) Representative images of γH2AX in irradiated IE1-expressing U2OS cells (Clone C102). Cells were treated as described in Fig. 2C. (C) Representative images of untreated U2OS PML$^{+/+}$ and $^{-/-}$ cells immunostained for PML. (D) Western blot analysis of U2OS cells transfected with untagged IE1 or an empty vector (EV). α-tubulin (Tub.) was used as a loading control. (E) Representative images of U2OS PML$^{+/+}$ and $^{-/-}$ cells transiently transfected with untagged IE1 or an empty vector (EV). Cells were irradiated with 1 Gy, fixed 1 h post irradiation, and immunostained for IE1 and γH2AX.

Data information: Scale bars, 5 μm.

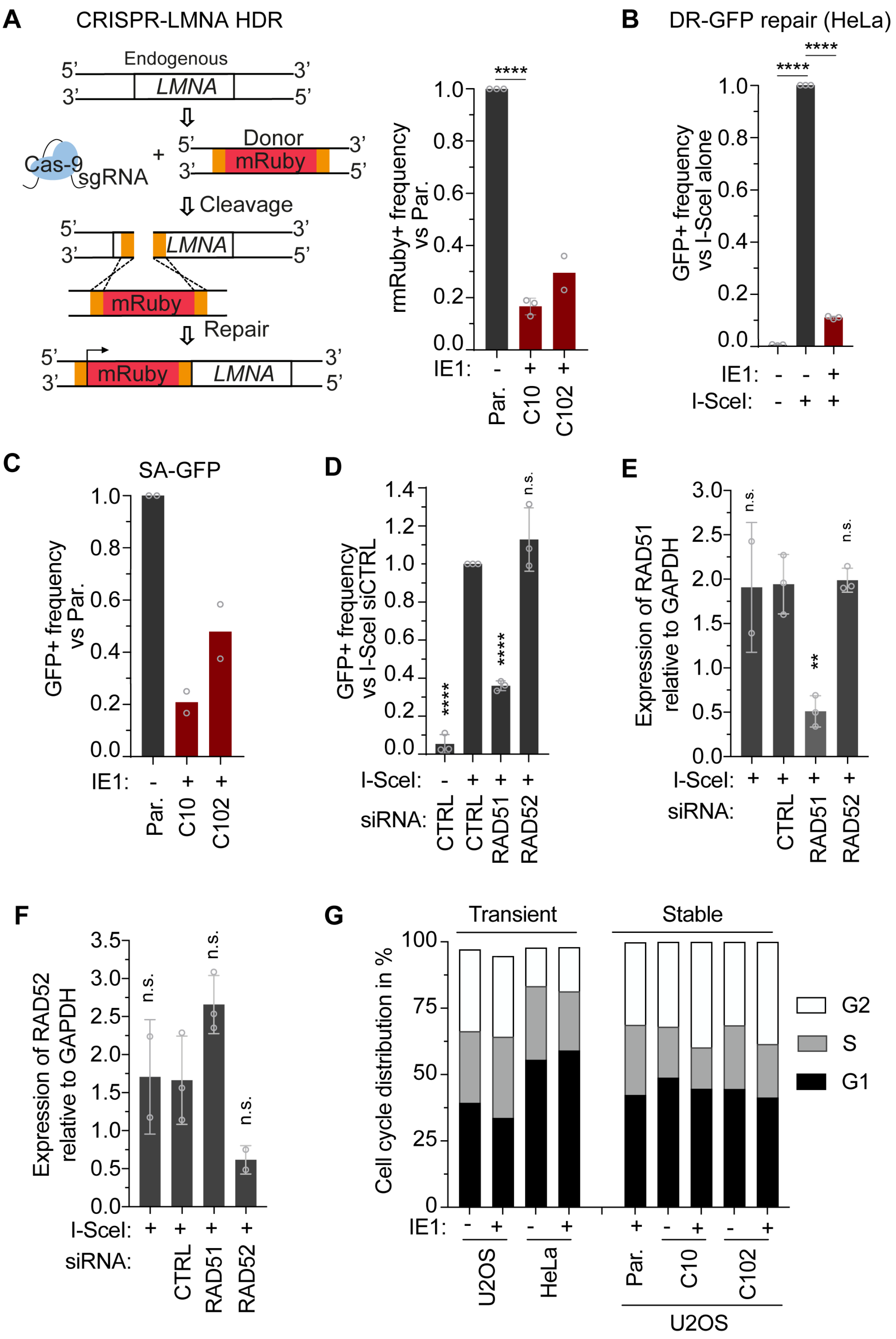
A
CRISPR-LMNA HDR
Endogenous
LMNA
Cas-9
sgRNA
Donor
mRuby
Cleavage
Repair
rmRuby+ frequency vs Par.
IE1:
Par.
C10
C102
B
DR-GFP repair (HeLa)
GFP+ frequency vs I-SceI alone
I-SceI:
C
SA-GFP
GFP+ frequency vs Par.
D
GFP+ frequency vs I-SceI siCTRL
siRNA:
CTRL
RAD51
RAD52
n.s.
E
Expression of RAD51 relative to GAPDH
F
Expression of RAD52 relative to GAPDH
G
Transient
Stable
Cell cycle distribution in %
G2
S
G1
U2OS
HeLa

Appendix Figure S3. Characterization of additional DNA repair reporter assays and associated controls in cells expressing IE1, related to Fig. 3.

(A) CRISPR-LMNA HDR assay in Dox-inducible IE1 U2OS SA-GFP stable cell lines. Cell lines were plated and induced for IE1 expression for 24 h prior to transfection with plasmids encoding Cas-9, a LMNA sgRNA, and the mRuby2-LMNA donor. The percentage of mRuby-positive cells was analyzed by flow cytometry 48 h post-transfection and normalized to the percentage of mRuby-positive U2OS SA cells (Par.) in each replicate (n ≥ 2 biological replicates). (B) DNA repair assays for homologous recombination in HeLa cells were performed as described for U2OS cells in Fig. 3A (n = 3 biological replicates). (C) SA-GFP assays were analyzed in Dox-inducible IE1 U2OS SA-GFP stable cell lines. Cell lines were plated and induced for IE1 expression for 24 h prior to transfection with a plasmid encoding I-Sce1 endonuclease. The percentage of GFP^+ cells was analyzed by flow cytometry 48 h post-transfection and normalized to the percentage of GFP-positive U2OS SA cells (Par.) in each replicate (n = 2 biological replicates). (D) Validation of the BIR repair assay using siRNAs against RAD51 and RAD52 (n = 3 biological replicates). A non-targeting siRNA (CTRL) was used as a control. (E-F) RAD51 (E) and RAD52 (F) RT-qPCR was performed on U2OS BIR cells (n ≥ 2 biological replicates). Expression was normalized against GADPH. (G) Cell cycle analyses of cells expressing IE1. The indicated cell lines were plated and either transfected or induced for IE1 expression for 24 h prior to fixation, PI staining, and flow cytometry (n = 1 biological replicates).

Data information: In (A, B, D, E, F), data are presented as mean ± SD, n.s. not significant, **p≤0.01, ****p≤0.0001 (One-way ANOVA with Dunnett's multiple comparison). In (D, E, F), the analyses were done by comparing with the conditions treated with the siCTRL. In (C, D), data are presented as mean ± SD.

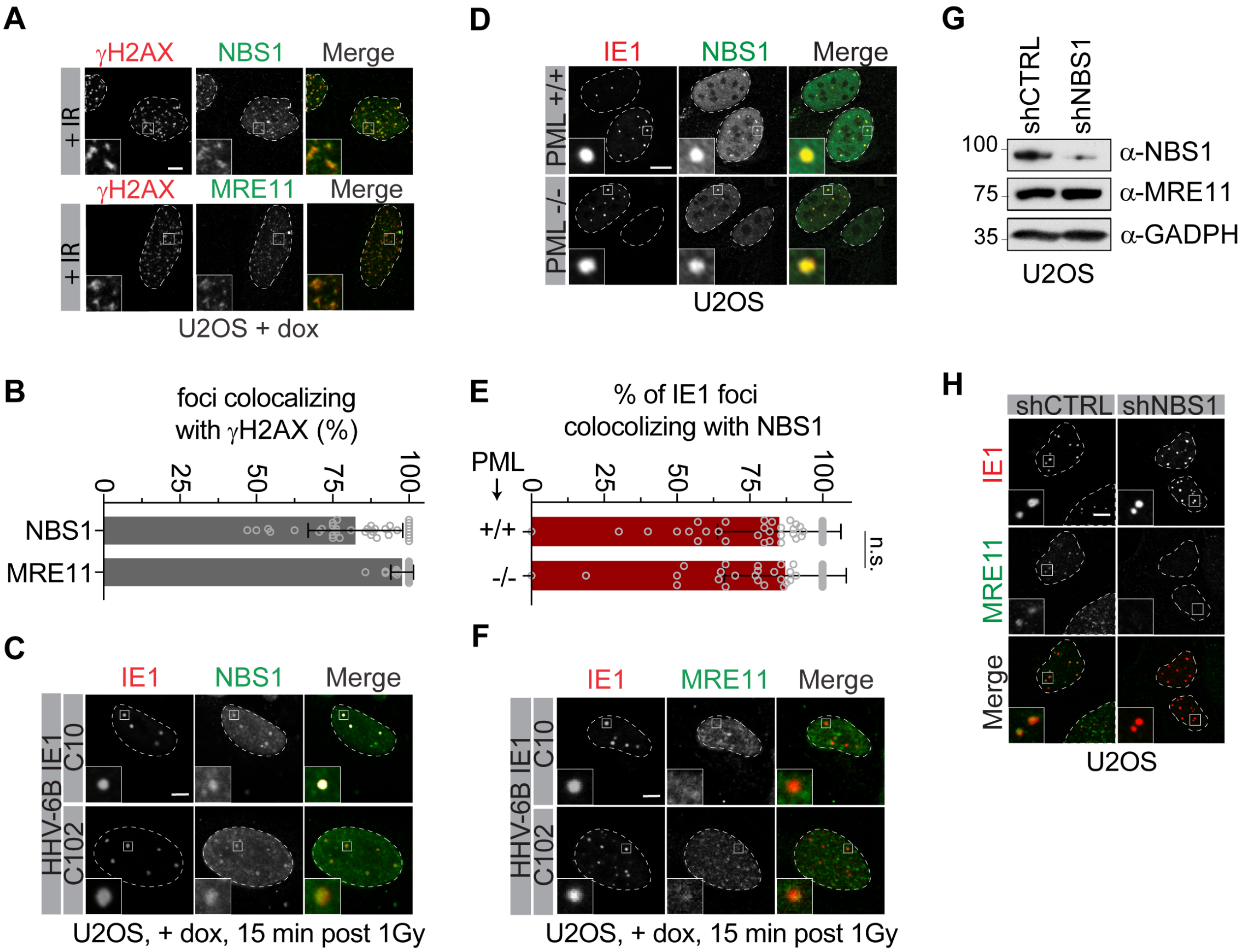

Appendix Figure S4. Representative images and analyses of the colocalization of IE1 with NBS1 in U2OS cells, related to Fig. 4.

(A) Representative images of the positive controls (irradiated cells) for Fig. 4C and E. Irradiated U2OS cells (+IR) were fixed 15 min post-irradiation (1 Gy) and immunostained for γH2AX, NBS1, or MRE11. (B) Quantification of γH2AX foci colocalized with NBS1 or MRE11 in irradiated cells, presented as a percentage of the total foci/cell colocalized with the indicated protein (n = 3 biological replicates). (C, F) Representative immunofluorescence of IE1-expressing U2OS stable cell lines induced with Dox and irradiated with 1 Gy. Cells were fixed 15 min post-irradiation and processed as described in Fig. 4A. (D-E) Representative immunofluorescence and quantification of IE1 foci that colocalize with NBS1 in U2OS PML$^{+/+}$ and $^{-/-}$ cells transiently expressing untagged IE1 (n = 3 biological replicates). (G) WCEs from U2OS shCTRL and shNBS1 stable cell lines were immunoblotted for NBS1 and MRE11. GAPDH was used as a loading control. (H) Representative images for the quantification presented in Fig. 4F.

Data information: In (E), data are presented as mean ± SD, n.s. not significant (unpaired t-test). In (B), data are presented as mean ± SD. Scale bars = 5 µm.

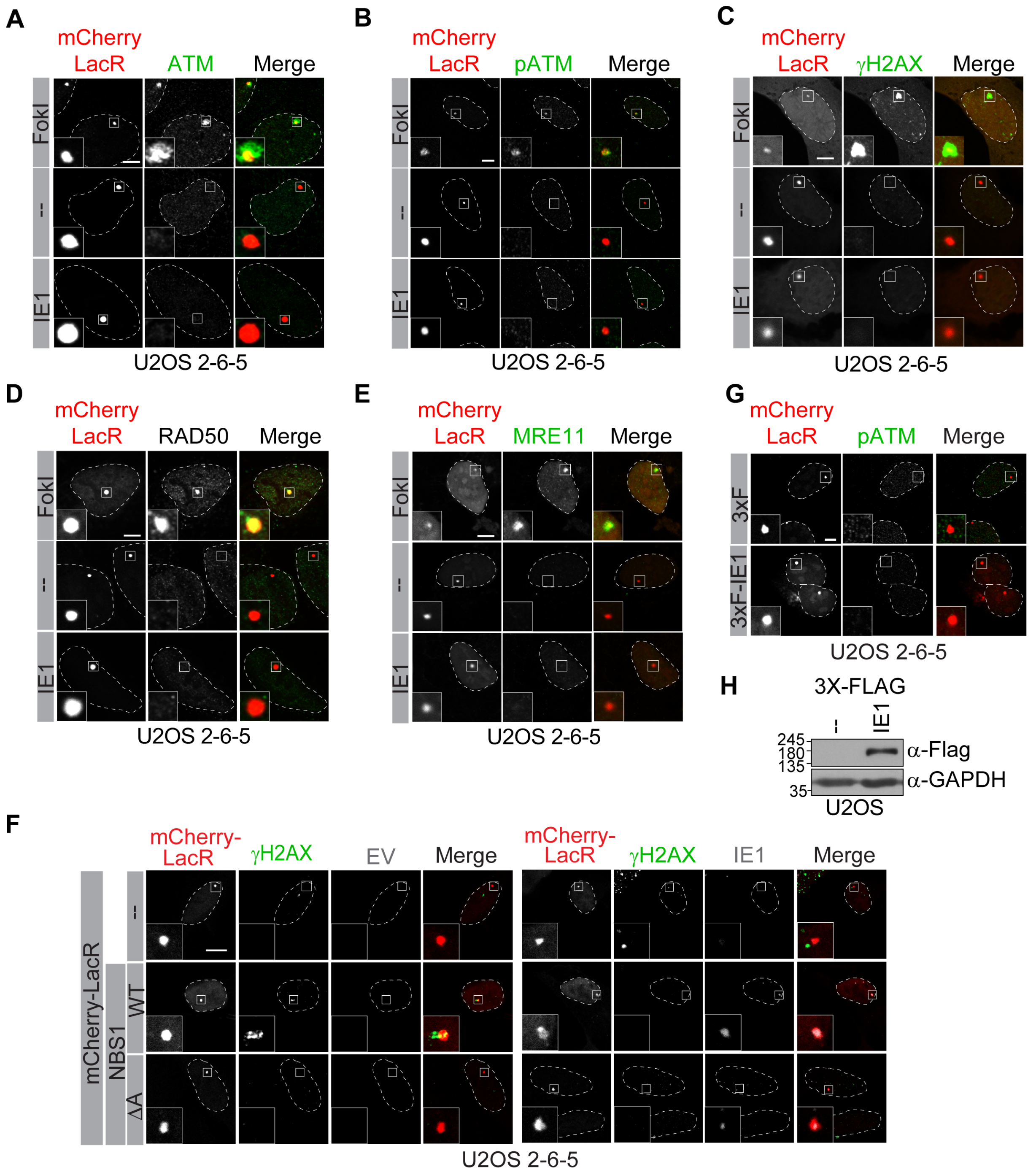

Appendix Figure S5. Representative image of the quantification presented in Fig. 5 and validation of Flag-IE1 expression in U2OS cells. (A-E) Representative images of the data presented in Fig. 5B. (F) Representative immunofluorescence used for the quantification presented in Fig. 5D. (G) Representative mCherry-LacR immunofluorescence used as a negative control in Fig. 5E. (H) WCEs from U2OS cells transiently transfected with the 3×FLAG-tagged IE1 expression vector or an empty vector were immunoblotted for FLAG. GAPDH was used as a loading control.

Data information: Scale bars = 5 µm.

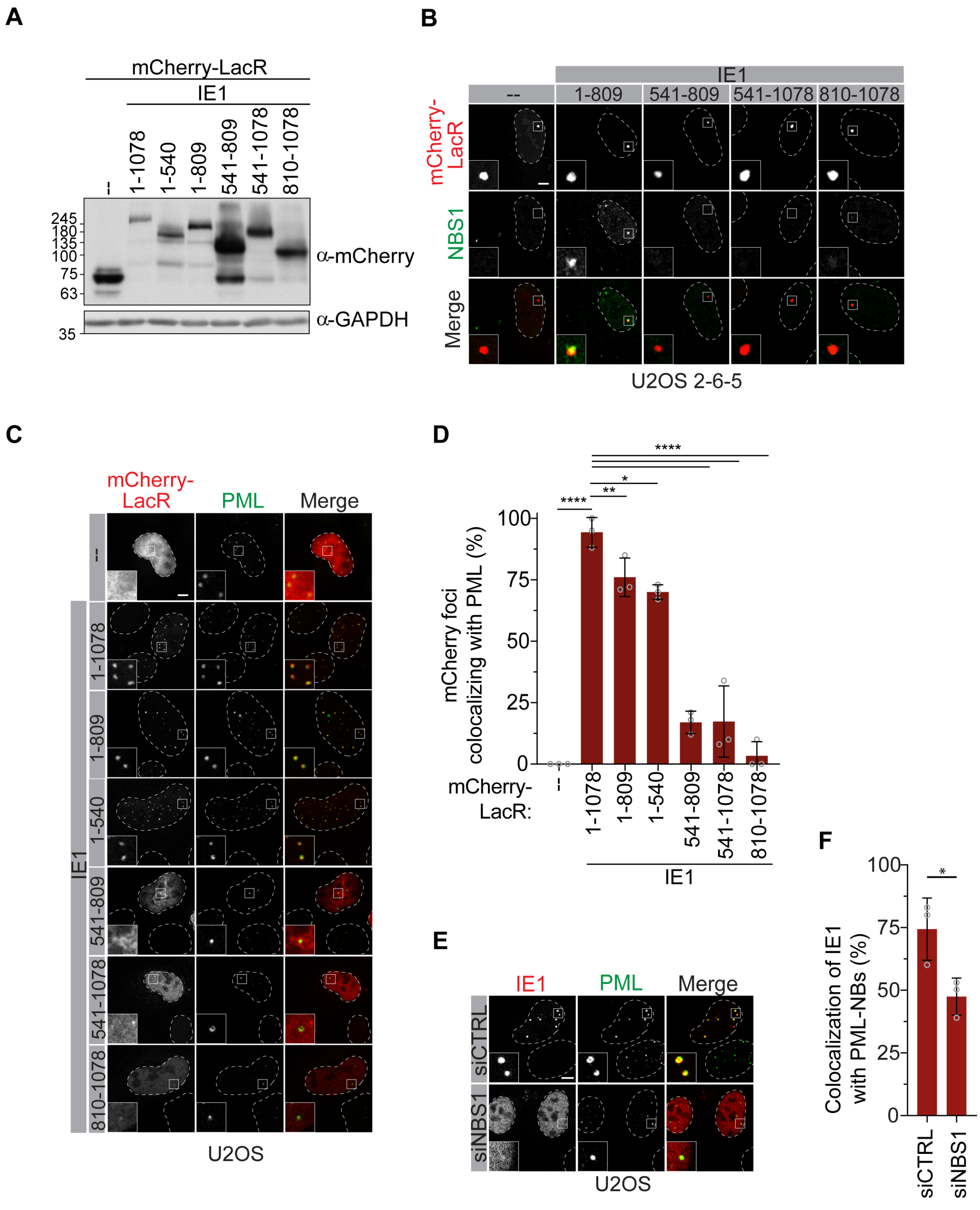
A
mCherry-LacR
IE1
--
1-1078
1-540
1-809
541-809
541-1078
810-1078
245
180
135
100
75
63
35
α-mCherry
α-GAPDH
B
IE1
--
1-809
541-809
541-1078
810-1078
mCherry-LacR
NBS1
Merge
U2OS 2-6-5
C
mCherry-LacR
PML
Merge
--
IE1
1-1078
1-809
1-540
541-809
541-1078
810-1078
U2OS
D
mCherry foci colocalizing with PML (%)
100
75
50
25
0
****
**
*
mCherry-LacR:
--
1-1078
1-809
1-540
541-809
541-1078
810-1078
IE1
E
IE1
PML
Merge
siCTRL
siNBS1
U2OS
F
Colocalization of IE1 with PML-NBs (%)
100
75
50
25
0
*
siCTRL
siNBS1

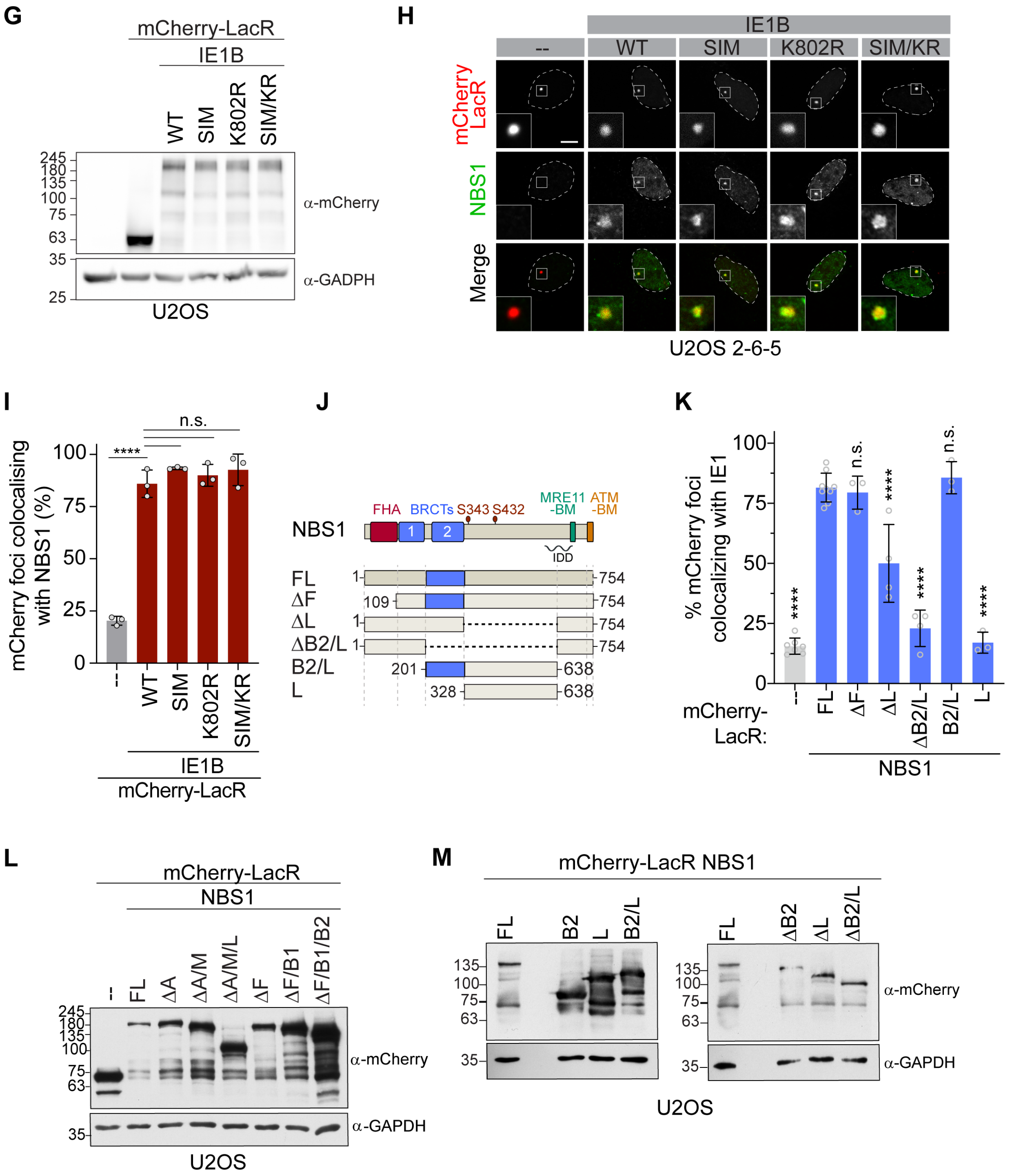
G
mCherry-LacR
IE1B
WT
SIM
K802R
SIM/KR
245
180
135
100
75
63
35
25
α-mCherry
α-GADPH
U2OS
H
IE1B
--
WT
SIM
K802R
SIM/KR
mCherry LacR
NBS1
Merge
U2OS 2-6-5
I
mCherry foci colocalising with NBS1 (%)
n.s.
****
100
75
50
25
0
--
WT
SIM
K802R
SIM/KR
IE1B
mCherry-LacR
J
NBS1
FHA
BRCTs
S343
S432
MRE11 -BM
ATM -BM
IDD
FL
ΔF
ΔL
ΔB2/L
B2/L
L
1
109
201
328
754
638
K
% mCherry foci colocalizing with IE1
100
75
50
25
0
n.s.
****
mCherry-LacR:
--
FL
ΔF
ΔL
ΔB2/L
B2/L
L
NBS1
L
mCherry-LacR
NBS1
--
FL
ΔA
ΔA/M
ΔA/M/L
ΔF
ΔF/B1
ΔF/B1/B2
245
180
135
100
75
63
35
α-mCherry
α-GAPDH
U2OS
M
mCherry-LacR NBS1
FL
B2
L
B2/L
ΔB2
ΔL
ΔB2/L
135
100
75
63
35
α-mCherry
α-GAPDH
U2OS

N

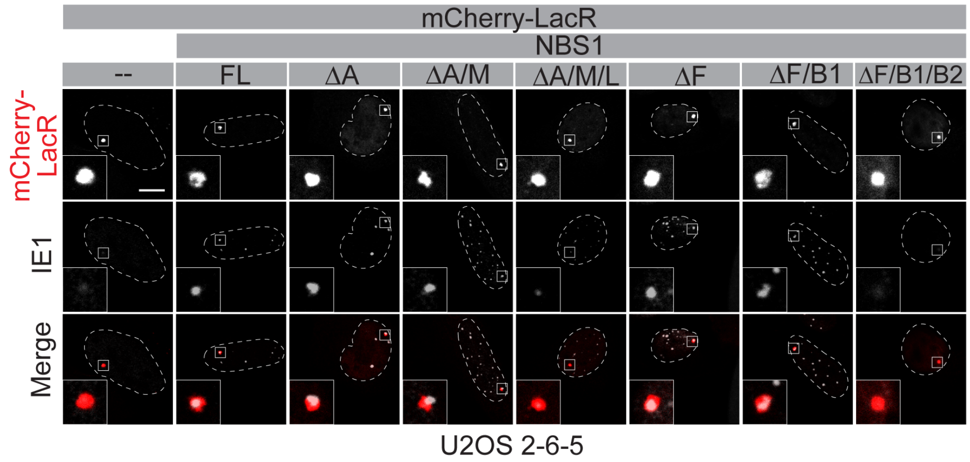

O

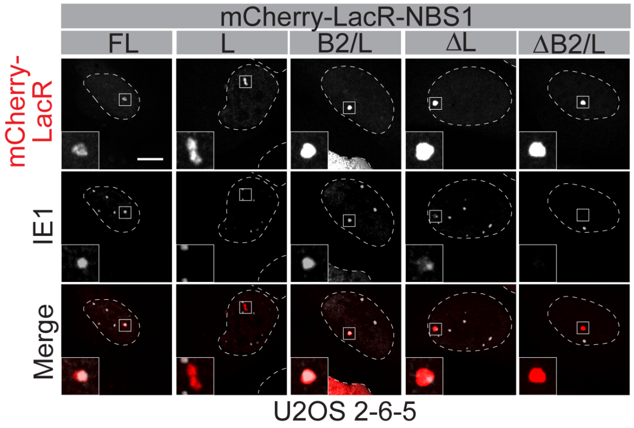

P

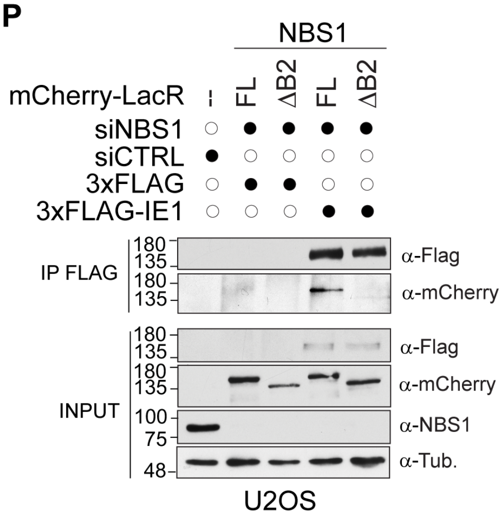

Appendix Figure S6. Validation of the expression and cellular localization of IE1 and NBS1 fragments used in this study, related to Fig. 6.

(A, G, L, M) WCEs from U2OS cells transiently transfected with the indicated mCherry-LacR or 3×FLAG fusion proteins and treated with the indicated siRNAs were immunoblotted for mCherry or FLAG. GAPDH was used as a loading control. The mCherry-LacR backbone was used as a negative control (--) in (A, G and L). (B, N, O) Representative images used for the quantifications presented in Fig. 6B (B), Fig. 6E (N, O), and SI Appendix, Fig. S6K (L, M). (C,D) Quantification and representative immunofluorescence of the minimal domain of mCherry-LacR-IE1 to be recruited to PML bodies. U2OS cells were transiently transfected with the indicated mCherry-LacR constructs and immunostained for PML (n = 3 biological replicates). (E, F) Quantification and representative immunofluorescence of the colocalization of IE1 with PML nuclear bodies (NBs) in NBS1-depleted cells. U2OS cells were treated with the indicated siRNAs, transfected with an IE1-expressing vector and immunostained for PML(n = 3 biological replicates). (H, I) Quantification and representative images of U2OS 2-6-5 cells transfected

with plasmids expressing the indicated mCherry-LacR fusion proteins were immunostained for NBS1 (n = 3 biological replicates). The mCherry-LacR backbone (--) alone was used as a negative control. (J) Schematic of NBS1 and the protein fragments used in this study. FHA, forkhead-associated domain; BRCT, BRCA1 C-terminal domain; MRE11-BM, MRE11-binding motif; ATM-BM, ATM-binding motif; IDD, intrinsically disordered domain. (K) Quantification of the colocalization of the indicated mCherry-LacR-NBS1 constructs with IE1 (n = 3 biological replicates). (P) U2OS cells treated with siCTRL or siNBS1 were transfected with the indicated 3×FLAG and mCherry-LacR constructs. After 24 h, WCEs were prepared and 3×-FLAG-IE1 interactors were immunoprecipitated using anti-Flag (M2) agarose beads and immunoblotted for FLAG, mCherry, and NBS1. α-tubulin (Tub.) was used as a loading control.

Data information: In (D, F, I, K), data are presented as mean ± SD, n.s. not significant, *p≤0.05, **p≤0.01, ****p≤0.0001 (D, I, K: One-way ANOVA with Dunnett's multiple comparison, F: unpaired t-test). Scale bars = 5 µm.

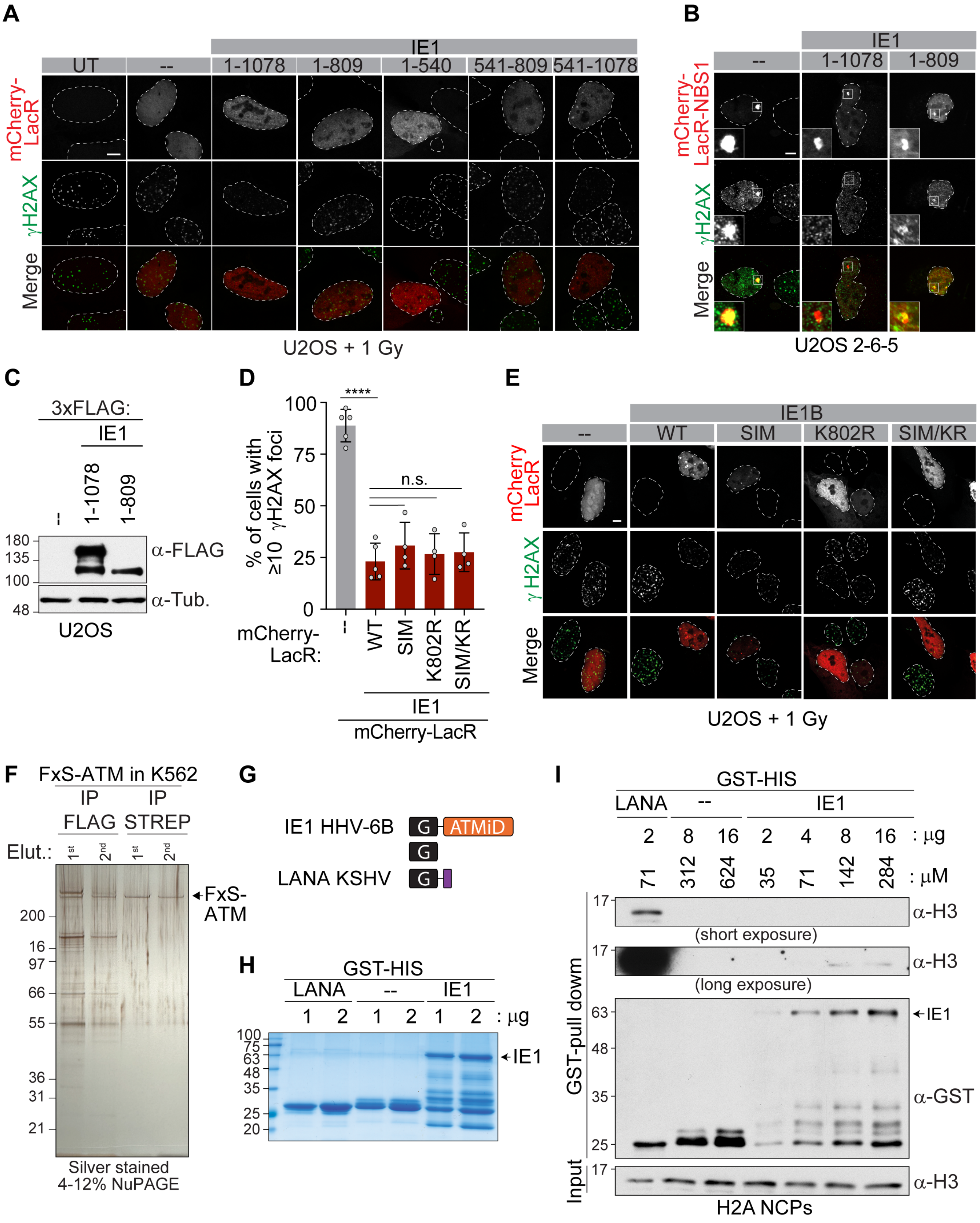
A
IE1
UT
--
1-1078
1-809
1-540
541-809
541-1078
mCherry-LacR
γH2AX
Merge
U2OS + 1 Gy
B
IE1
--
1-1078
1-809
mCherry-LacR-NBS1
γH2AX
Merge
U2OS 2-6-5
C
3xFLAG:
IE1
--
1-1078
1-809
180
135
100
48
α-FLAG
α-Tub.
U2OS
D
****
n.s.
% of cells with ≥10 γH2AX foci
100
75
50
25
0
mCherry-LacR:
--
WT
SIM
K802R
SIM/KR
IE1
mCherry-LacR
E
IE1B
--
WT
SIM
K802R
SIM/KR
mCherry LacR
γH2AX
Merge
U2OS + 1 Gy
F
FxS-ATM in K562
IP FLAG
IP STREP
Elut.:
1st
2nd
1st
2nd
FxS-ATM
200
16
97
66
55
36
31
21
Silver stained
4-12% NuPAGE
G
IE1 HHV-6B
G
ATMiD
G
LANA KSHV
G
H
GST-HIS
LANA
--
IE1
1 2 1 2 1 2 : µg
100
75
63
48
35
25
20
IE1
I
GST-HIS
LANA
--
IE1
2 8 16 2 4 8 16 : µg
71 312 624 35 71 142 284 : µM
17
α-H3
(short exposure)
17
α-H3
(long exposure)
63
48
35
25
IE1
α-GST
GST-pull down
17
Input
α-H3
H2A NCPs

Appendix Figure S7. Validation of the expression and cellular localization of IE1 fragments used in to study the inhibition of ATM by IE1, related to Fig. 7.

(A, B, E) Representative images used for the quantifications presented in Fig. 7B (A), Fig. 7D (B), and Appendix Fig. S7D (E). (C) WCEs from U2OS cells transiently transfected with the indicated 3×FLAG fusion proteins were immunoblotted for FLAG. Tubulin was used as a loading control. The 3xFLAG backbone was used as a negative control (--). (D) Quantification of U2OS cells transfected with plasmids expressing the indicated mCherry-LacR fusion protein and irradiated (1 Gy) were immunostained for γH2AX (n = 3 biological replicates). The 3xFLAG backbone (--) alone was used as a negative control. (F) Silver-stained SDS-PAGE showing the purified native ATM tandem affinity purification. A clonal K562 cell line expressing 3xFLAG-2xStrep tagged ATM (ATM), from the AAVS1 safe harbor were used for the purification as described (Dalvai *et al*, 2015). Eluted fractions (elution 1st and 2nd) from the two purification steps are shown. (G) Schematic of HHV-6B IE1 ATMiD and KSHV LANA recombinant proteins that are used in I. (H) Coomassie Brilliant Blue (CBB) staining of the indicated recombinant GST-HIS-tagged proteins. (I) Pull down assays of 0.75 µg of nucleosome core particles (NCPs) with the indicated GST fusion proteins. KSHV LANA peptide is used as a positive control. Amount of protein used in each pull down is indicated in µg as well as in µM for comparison.

Data information: In (D), data are presented as mean ± SD, n.s. not significant, ****p≤0.0001 (One-way ANOVA with Dunnett's multiple comparison). Scale bars = 5 µm.

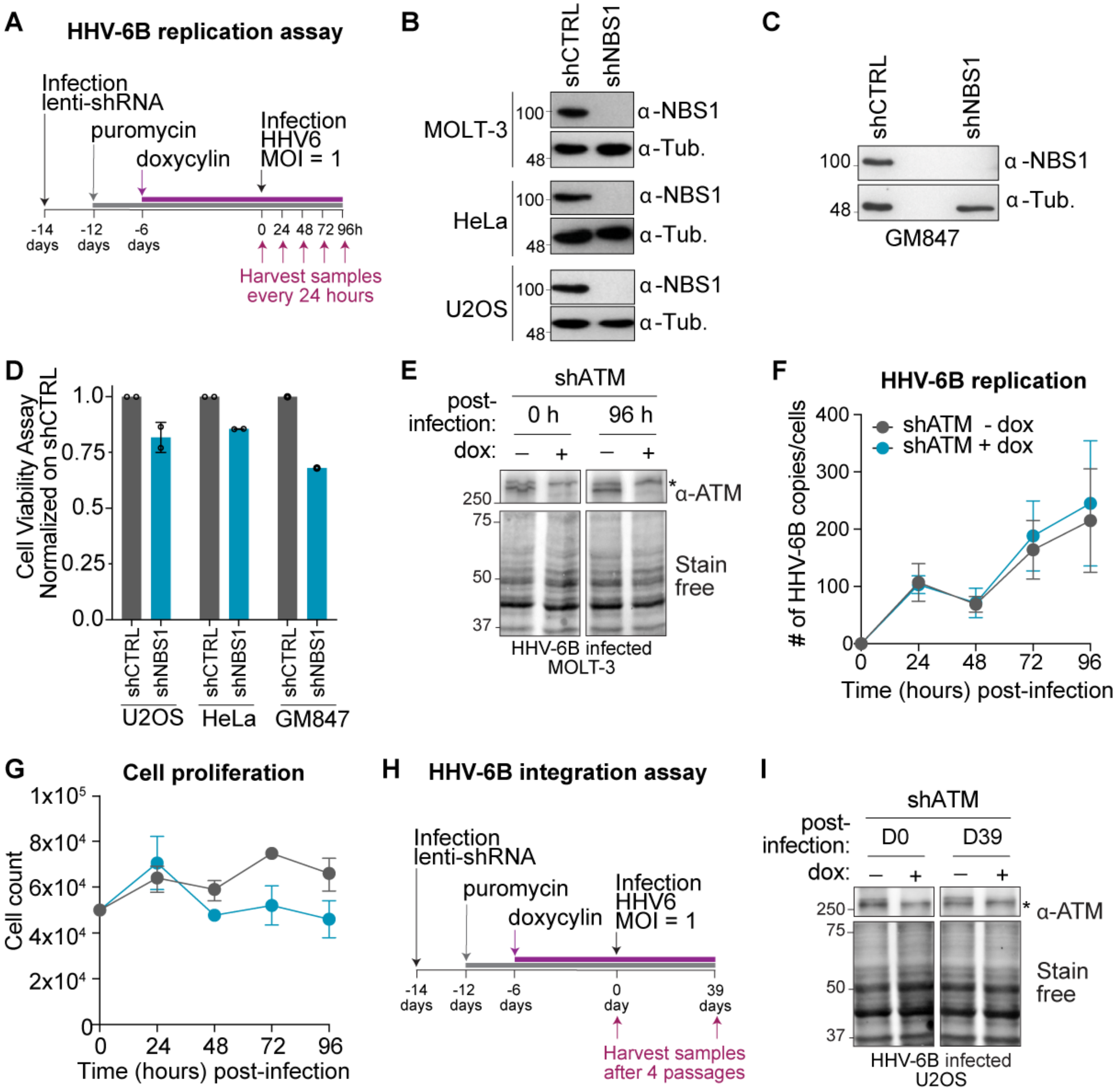

J

Table R1. Importance of ATM for HHV-6B chromosomal integration in ALT+ cells

Cell line	ALT status	shRNA	% cells with integrated HHV-6B[a] (n)[b]	*P* value[a]
U2OS	Positive	ATM^{-dox}	0,72 (18,052)	n.s.[b]
		ATM^{+dox}	0,79 (11,760)	

[a] *Pearson's Chi-squared test with Yates' continuity correction*
[b] *ns, not significant*

Appendix Figure S8. Validation of NBS1 and ATM knockdown and its impact on cell viability.
(A, H) Timeline of the experiments that were done to investigate HHV-6B replication and integration. (B, C, E, I) WCEs from the indicated cells lines expressing an shRNA against NBS1 (shNBS1), a control shRNA (shCTRL) or a shRNA against ATM (shATM in E, 0h, 96h, and in I Day 0 and Day39) were immunoblotted for NBS1 or ATM. α-Tubulin (Tub.) or Stain-free signal (Bio-Rad Stain-Free) polyacrylamide gels was used as a loading control. Asterisk (*) in (E) indicated a non-specific band. (D) Quantification of cell viability by CellTiter-Glo® assay. The numbers of viable cells were determined using standard curves for each cell line, with the number of cells in the shCTRL sample set to 1.0 (n = 1 and n = 2 biological replicates). (F) MOLT-3 cells with and without the induction of shATM were infected with HHV-6B at a MOI of 1 and harvested at the indicated time points. Following cell lysis, DNA was extracted and HHV-6B was quantified by ddPCR using primers for HHV-6B U67-68 and human RPP30 (n ≥ 3 biological replicates). (G) Cell proliferation was quantified by counting cells at the indicated time points (n = 3 biological replicates). Percent of cells in which the viral DNA is integrated is reported in the table presented in (J).

Data information: In (D, F, G), data are presented as mean ± SD. In (J), data were analyzed using Pearson's Chi-squared test with Yates' continuity correction. n.s. not significant.

Appendix table S1. Plasmids used in this study.

Plasmid	Source
pcDNA4/TO/myc-His-HHV-6B IE1	Previously described (Jaworska *et al*, 2007)
pcDNA4/TO/myc-His	ThermoFisher, V103020
pDEST-PB-TetO	Previously described (Ho *et al*, 2018)
PB-CA-rtTA-IRES-NEO	Previously described (Ho *et al*, 2018)
PB-TetO-HHV-6B IE1	This study
pDEST-mCherry-LacRnls	Previously described (Orthwein *et al*, 2015)
mCherry-LacRnls HHV-6B IE1 1-1078	This study
mCherry-LacRnls HHV-6B IE1 1-809	This study
mCherry-LacRnls HHV-6B IE1 1-540	This study
mCherry-LacRnls HHV-6B IE1 541-809	This study
mCherry-LacRnls HHV-6B IE1 541-1078	This study
mCherry-LacRnls HHV-6B IE1 810-1078	This study
pDEST-FRT-TO-3×FLAG	Gift from Anne-Claude-Gingras (V4978) (Couzens *et al*, 2013)
3×FLAG HHV-6B IE1 1-1078	This study
3×FLAG HHV-6B IE1 1-809	This study
mCherry-LacRnls NBS1 1-754 (WT)	This study
mCherry-LacRnls NBS1 1-733 (ΔA)	This study
mCherry-LacRnls NBS1 1-638 (ΔA/ΔM)	This study
mCherry-LacRnls NBS1 1-327 (ΔA/ΔM/ΔL)	This study
mCherry-LacRnls NBS1 109-754 (ΔF)	This study
mCherry-LacRnls NBS1 201-754 (ΔF/ΔB1)	This study
mCherry-LacRnls NBS1 328-754 (ΔF/ΔB1/ΔB2)	This study
mCherry-LacRnls NBS1 Δ201-638 (ΔB2/L)	This study
mCherry-LacRnls NBS1 Δ201-327 (ΔB2)	This study
mCherry-LacRnls NBS1 Δ328-638 (ΔL)	This study
mCherry-LacRnls NBS1 201-327 (B2)	This study
mCherry-LacRnls NBS1 328-638 (L)	This study
mCherry-LacRnls NBS1 201-638 (B2/L)	This study
pLXIN2-NBS1	Gift from Cary A. Moody (Anacker *et al*, 2014)
pLKO.1-Scramble	Addgene 136035
pLKO.1-shNBS1	Gift from Cary A. Moody (Anacker *et al*, 2014)
pMD2.g	Addgene 12259
psPAX	Addgene 12260
pBIR-GFP	Addgene 49807
pCBASceI	Addgene 26477
pX330-LMNA-gRNA1	Gift from Jean-Yves Masson
pCR2.a-mRuby2-LMNA-Donor	Gift from Jean-Yves Masson

pX330-7a sgRNA (EJ7-GFP reporter)	Addgene 113620
pX330-7b sgRNA (EJ7-GFP reporter)	Addgene 113624
pLenti X2 Hygro/pTER shATM #2 (w639-1)	Addgene 22532
pETM-30 (GST-His)	Gift from Daniel Durocher (Fradet-Turcotte *et al*, 2013)
pETM-30 (GST-His) LANA	Gift from Daniel Durocher (Fradet-Turcotte *et al*, 2013)
pETM-30 (GST-His) HHV-6B IE1 810-1078	This study

Appendix table S2. Primers, siRNA, shRNAs, and FISH probe sequences used in this study.

Name	Sequence (5′-3′)	Purpose
shCTRL<br>*(Sigma: SHC202)*	CCGGCAACAAGATGAAGAGCACCAACTCGAGTTGG TGCTCTTCATCTTGTTGTTTTT	Control shRNA in U2OS, MOLT-3, HeLa, GM847 cells
shNBS1<br>*(TRCN0000010393)*	GAAGAGTGGCTAAGGCAGGAA	KD NBS1 in U2OS, MOLT-3, HeLa, GM847 cells
shATM #2<br>*(Addgene 22532)*	GATCCCTGAAGATGGTGCTCATAAATTCAAGAGATT TATGAGCACCATCTTCATTTTTGGAAA	KD ATM in MOLT-3 cells
U67-68 FW	TTCCGGTATATGACCTTCGTAAGC	ddPCR
U67-68 RV	GATGTCTCACCTCCAAATCTTTAGAAAT	ddPCR
U67-68 Probe	FAM-ACATTATAT-ZEN-RTCGAAYYTGACR CTACCTTCCG-IABkFQ<br>*Mixed probe where R = A or G, Y = C or T to accommodate the sequences of HHV-6A and -6B*	ddPCR[c]
RPP30 FW	GATTTGGACCTGCGAGCG	ddPCR
RPP30 RV	GCGGCTGTCTCCACAAGT	ddPCR
RPP30 Probe	HEX-TCTGACCTG-ZEN-AAGGCTCTGCGCG-IABkFQ	ddPCR[c]
GAPDH FW	CAACGTGTCAGTGGTGGACC	RT, qPCR[b]
GAPDH RV	TCGTTGAGGGCAATGCCAGC	RT, qPCR[b]
RAD51 FW	TCTCTGGCAGTGATGTCCTGGA	qPCR
RAD51 RV	TAAAGGGCGGTGGCACTGTCTA	qPCR
RAD52 FW	ATGCTTTGGACAGTGCCAGT	qPCR
RAD52 RV	CCAGCCATGCGGCTACTTAT	qPCR
siRAD52<br>*(custom)*	GGCUACAAUCUUCAUUUAAdTdT	KD in U2OS BIR assays
siRAD51<br>*(M-003530-04-0005)*	Smartpool:<br>D-003530-02: GAAGCUAUGUUCGCCAUUA<br>D-003530-05: GCAGUGAUGUCCUGGAUAA<br>D-003530-07: CCAACGAUGUGAAGAAAUU	KD in U2OS BIR assays

	D-003530-08: AAGCUAUGUUCGCCAUUAA	
siNBS1 (custom)	AACAUACGUAGCUGACACAGAdTdT	KD in U2OS immunoprecipitation/ immunostaining
siCTRL *(D-001210-02-02)*	UAAGGCUAUGAAGAGAUAC	CTRL in U2OS BIR assays
Cy5-TelC PNA probe *(F1003)*	[Cy5]-OO-CCCTAACCCTAACCCTAA	FISH

[a] KD: knockdown, gDNA: genomic DNA
[b] Used to validate the efficiency of DNAse treatment prior to RT-PCR
[c] FAM: fluorescein, HEX: HEX fluorophore, ZEN: ZEN[TM] quencher, IABkFQ: 3′ Iowa Black® forward quencher

Appendix table S3. Antibodies used in this study.

**Primary antibodies**		
**Target**	**Catalog number (Source)**	**Purpose[a]**
Mouse anti-phospho-Histone H2A.X (Ser139) (γH2AX), clone JBW30	05-636 (Millipore) RRID:AB_309864	IF
Rabbit anti-phospho-Histone H2A.X (Ser139) (γH2AX)	2577L (Cell Signaling Technology) RRID:AB_2118010	IF, IB
Mouse anti-phospho-Histone H2A.X (Ser139) (γH2AX), clone JBW30, alexa fluor conjugate	05-636-AF555 (Millipore)	IF
Mouse anti-α-tubulin	CP06 (Sigma) RRID:AB_2617116	IB
Mouse anti-GAPDH (ZG003)	39-8600 (ThermoFisher) RRID:AB_2533438	IB
Rabbit anti-IE1	Homemade(Gravel *et al*, 2002)	IF, IB
Rabbit anti-IE1, alexa fluor conjugate	Homemade(Gravel *et al*, 2002)	IF
Rabbit anti-lamin B1	ab16048 (Abcam) RRID:AB_443298	IF
Human CREST anti-centromere serum	HCT-0100 (Immunovision) RRID:AB_2744669	IF
Mouse anti-PML (PG-M3)	sc-966 (Santa Cruz Biotechnology) RRID:AB_628162	IF
Mouse anti-NBS1 1D7	GTX70224 (GeneTex) AB_372445	IF, IB
Mouse anti-MRE11 12D7	GTX70212 (GeneTex) AB_372398	IF, IB
Mouse anti-RAD50 13D3	GTX70228 (GeneTex) AB_372854	IF, IB
Rabbit anti-FLAG	2368S (Cell Signaling Technology) RRID:AB_2217020	IF,
Mouse anti-FLAG M2	F1804 (Millipore Sigma) AB_262044	IF, IB, IP
Rabbit anti-ATM	A300-299A (Bethyl Laboratories) RRID:AB_263415	IF, IB
Rabbit anti-pATM (S1981) D25E5	13050 (Cell Signaling Technology) RRID:AB_2798100	IF, IB
Rabbit anti-mCherry	NBP2-25157 (Novus)	IB

	RRID:AB_2753204	
Rabbit anti-H3	ab1791 (Abcam)<br>RRID:AB_302613	IB
Rabbit anti-GST (Z-5)	SC-459 (Santa Cruz)<br>RRID:AB_631586	IB
**Secondary antibodies**		
**Target**	**Catalog number (Source)**	**Purpose[a]**
Alexa Fluor 488 goat anti-rabbit	A-11034 (Thermo Fisher Scientific)<br>RRID:AB_2576217	IF
Alexa Fluor 555 goat anti-rabbit	A-21428 (Thermo Fisher Scientific)<br>RRID:AB_141784	IF
Alexa Fluor 647 goat anti-rabbit	A-21244 (Thermo Fisher Scientific)<br>RRID:AB_2535812	IF
Alexa Fluor 488 goat anti-mouse	A-11029 (Thermo Fisher Scientific)<br>RRID:AB_2534088	IF
Alexa Fluor 555 goat anti-mouse	A-21424 (Thermo Fisher Scientific)<br>RRID:AB_141780	IF
Alexa Fluor 647 goat anti-mouse	A-21236 (Thermo Fisher Scientific)<br>RRID:AB_2535805	IF
Alexa Fluor 647 donkey anti-human	A-21445 (Thermo Fisher Scientific)<br>RRID:AB_2340577	IF
Goat anti-rabbit HRP	7074 (Cell Signaling Technology)<br>RRID:AB_2307391	IB
Sheep anti-mouse-HRP	A-9044 (Sigma)<br>RRID:AB_258431	IB

[a] IF: immunofluorescence, IB: immunoblotting, IP: immunoprecipitation

Appendix References

Anacker DC, Gautam D, Gillespie KA, Chappell WH & Moody CA (2014) Productive Replication of Human Papillomavirus 31 Requires DNA Repair Factor Nbs1. *J Virol* 88: 8528–8544

Boonen RACM, Rodrigue A, Stoepker C, Wiegant WW, Vroling B, Sharma M, Rother MB, Celosse N, Vreeswijk MPG, Couch F, *et al* (2019) Functional analysis of genetic variants in the high-risk breast cancer susceptibility gene PALB2. *Nat Commun* 10

Couzens AL, Knight JDR, Kean MJ, Teo G, Weiss A, Dunham WH, Lin ZY, Bagshaw RD, Sicheri F, Pawson T, *et al* (2013) Protein interaction network of the mammalian hippo pathway reveals mechanisms of kinase-phosphatase interactions. *Sci Signal* 6

Dalvai M, Loehr J, Jacquet K, Huard CC, Roques C, Herst P, Côté J & Doyon Y (2015) A Scalable Genome-Editing-Based Approach for Mapping Multiprotein Complexes in Human Cells. *Cell Rep* 13

Fradet-Turcotte A, Canny MDMD, Escribano-Díaz C, Orthwein A, Leung CCYCCY, Huang H, Landry M-CMC, Kitevski-Leblanc J, Noordermeer SMSM, Sicheri F, *et al* (2013) 53BP1 is a reader of the DNA-damage-induced H2A Lys 15 ubiquitin mark. *Nature* 499

Gravel A, Gosselin J & Flamand L (2002) Human herpesvirus 6 immediate-early 1 protein is a sumoylated nuclear phosphoprotein colocalizing with promyelocytic leukemia protein-associated nuclear bodies. *Journal of Biological Chemistry* 277: 19679–19687

Ho T-H, Sitz J, Shen Q, Leblanc-Lacroix A, Campos EI, Borozan I, Marcon E, Greenblatt J, Fradet-Turcotte A, Jin D-Y, *et al* (2018) A Screen for Epstein-Barr Virus Proteins That Inhibit the DNA Damage Response Reveals a Novel Histone Binding Protein. *J Virol* 92

Jaworska J, Gravel A, Fink K, Grandvaux N & Flamand L (2007) Inhibition of Transcription of the Beta Interferon Gene by the Human Herpesvirus 6 Immediate-Early 1 Protein. *J Virol* 81: 5737–5748

Orthwein A, Noordermeer SM, Wilson MD, Landry S, Enchev RI, Sherker A, Munro M, Pinder J, Salsman J, Dellaire G, *et al* (2015) A mechanism for the suppression of homologous recombination in G1 cells. *Nature* 528: 422–426

Sitz J, Blanchet SA, Gameiro SF, Biquand E, Morgan TM, Galloy M, Dessapt J, Lavoie EG, Blondeau A, Smith BC, *et al* (2019) Human papillomavirus E7 oncoprotein targets RNF168 to hijack the host DNA damage response. *Proc Natl Acad Sci U S A* 116

Tang J, Cho NW, Cui G, Manion EM, Shanbhag NM, Botuyan MV, Mer G & Greenberg RA (2013) Acetylation limits 53BP1 association with damaged chromatin to promote homologous recombination. *Nat Struct Mol Biol* 20: 317–325
