# Supplementary figures and images for: The immediate-early protein 1 of human herpesvirus 6B interacts with NBS1 and inhibits ATM signaling
Source: EMBO Rep. 2024 Jan 2;25(2):725–44. doi: 10.1038/s44319-023-00035-z (PMC10897193; doi:10.1038/s44319-023-00035-z)

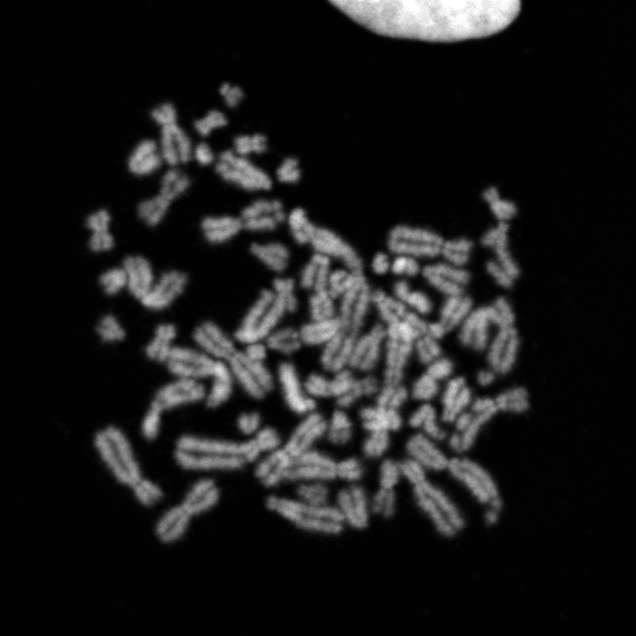

Supplement: Supplementary file 3 — Source Data Fig. 1 [file 44319_2023_35_MOESM3_ESM.zip › Figure 1/1F/image data_HHV-6B IE1 C10.lsm]

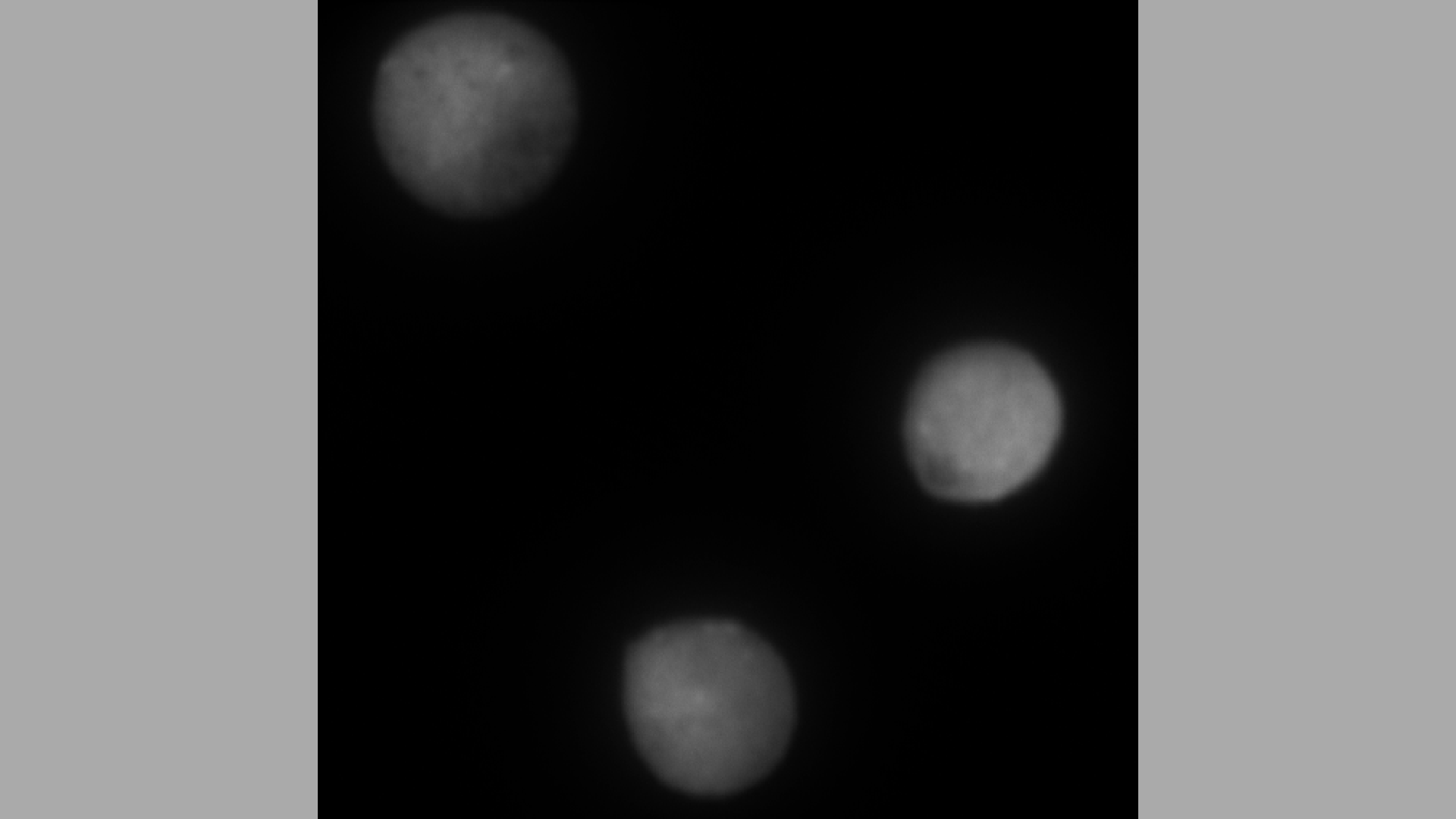

Supplement: Supplementary file 3 — Source Data Fig. 1 [file 44319_2023_35_MOESM3_ESM.zip › Figure 1/1A/image data_Mock.tif]

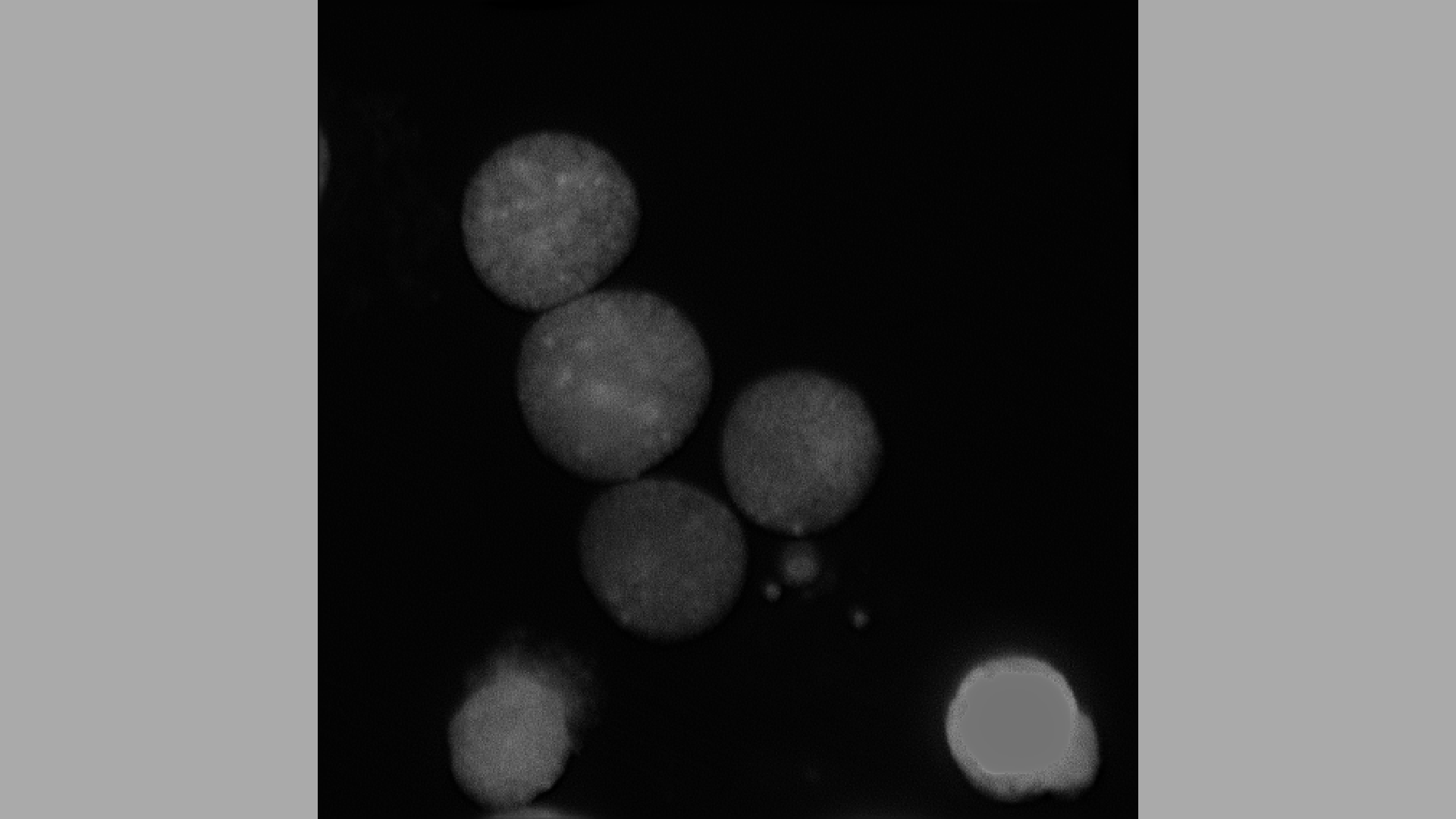

Supplement: Supplementary file 3 — Source Data Fig. 1 [file 44319_2023_35_MOESM3_ESM.zip › Figure 1/1A/image data_HHV-6B.tif]

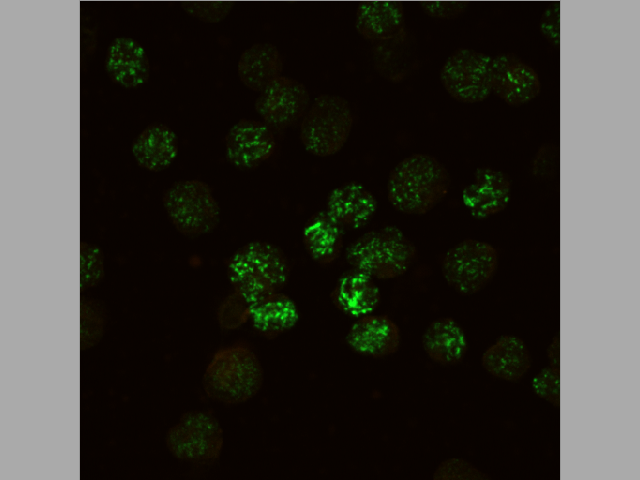

Supplement: Supplementary file 4 — Source Data Fig. 2 [file 44319_2023_35_MOESM4_ESM.zip › Figure 2/2A/image data_MOLT3 Mock-MERGE.tif]

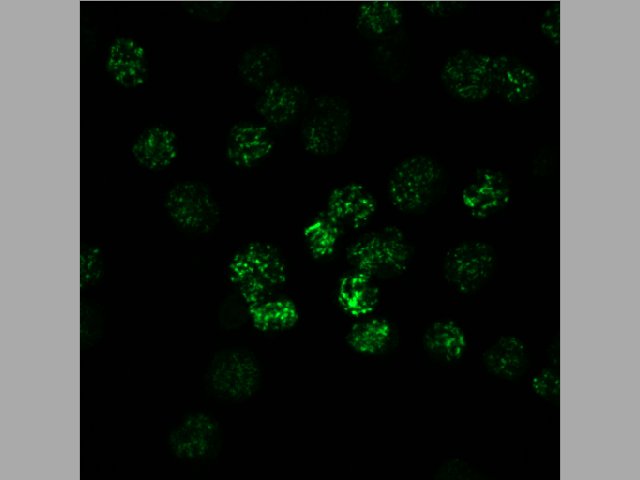

Supplement: Supplementary file 4 — Source Data Fig. 2 [file 44319_2023_35_MOESM4_ESM.zip › Figure 2/2A/image data_MOLT3 Mock-gH2AX.tif]

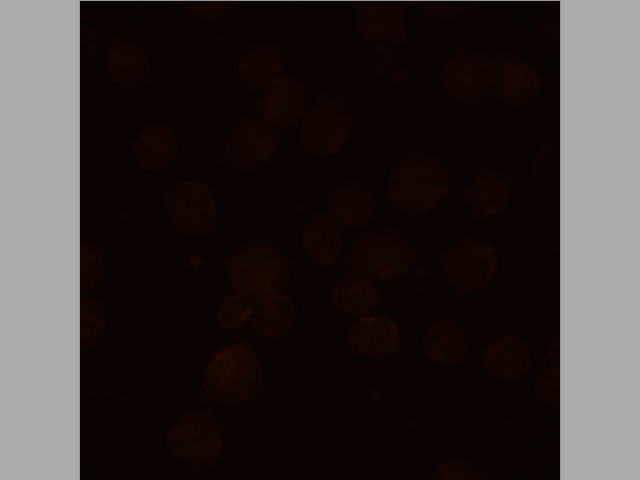

Supplement: Supplementary file 4 — Source Data Fig. 2 [file 44319_2023_35_MOESM4_ESM.zip › Figure 2/2A/image data_MOLT3 Mock-IE1.tif]

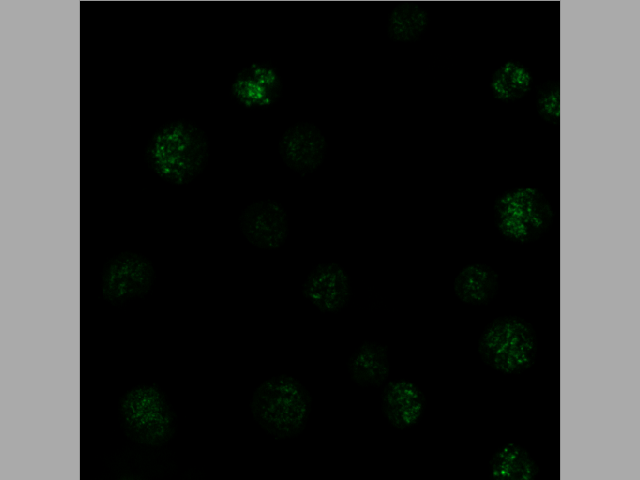

Supplement: Supplementary file 4 — Source Data Fig. 2 [file 44319_2023_35_MOESM4_ESM.zip › Figure 2/2A/image data_MOLT3 HHV-6B- gH2AX.tif]

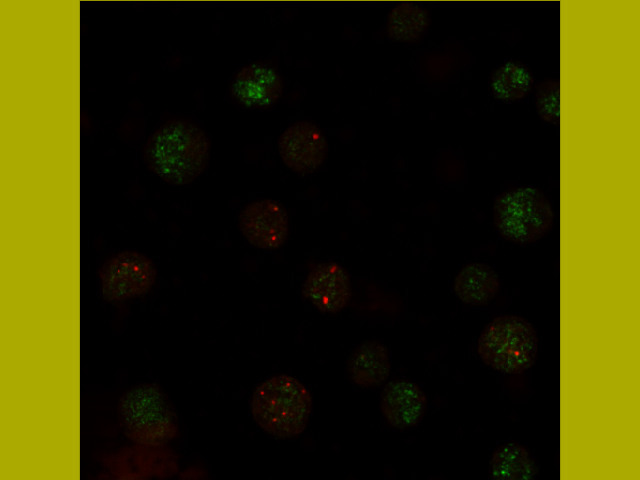

Supplement: Supplementary file 4 — Source Data Fig. 2 [file 44319_2023_35_MOESM4_ESM.zip › Figure 2/2A/image data_MOLT3 HHV-6B-MERGE.tif]

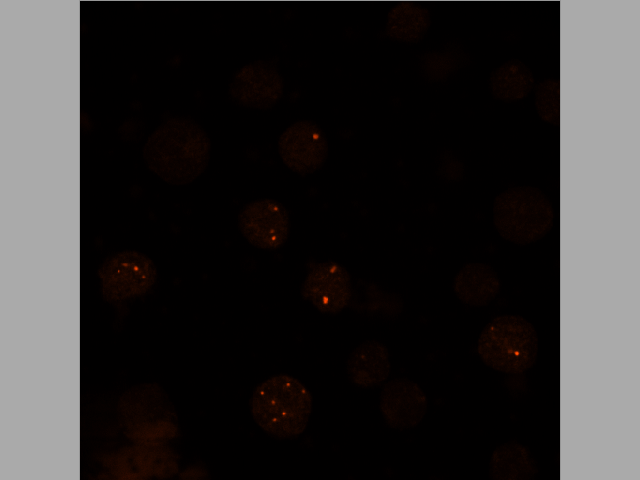

Supplement: Supplementary file 4 — Source Data Fig. 2 [file 44319_2023_35_MOESM4_ESM.zip › Figure 2/2A/image data_MOLT3 HHV-6B-IE1.tif]

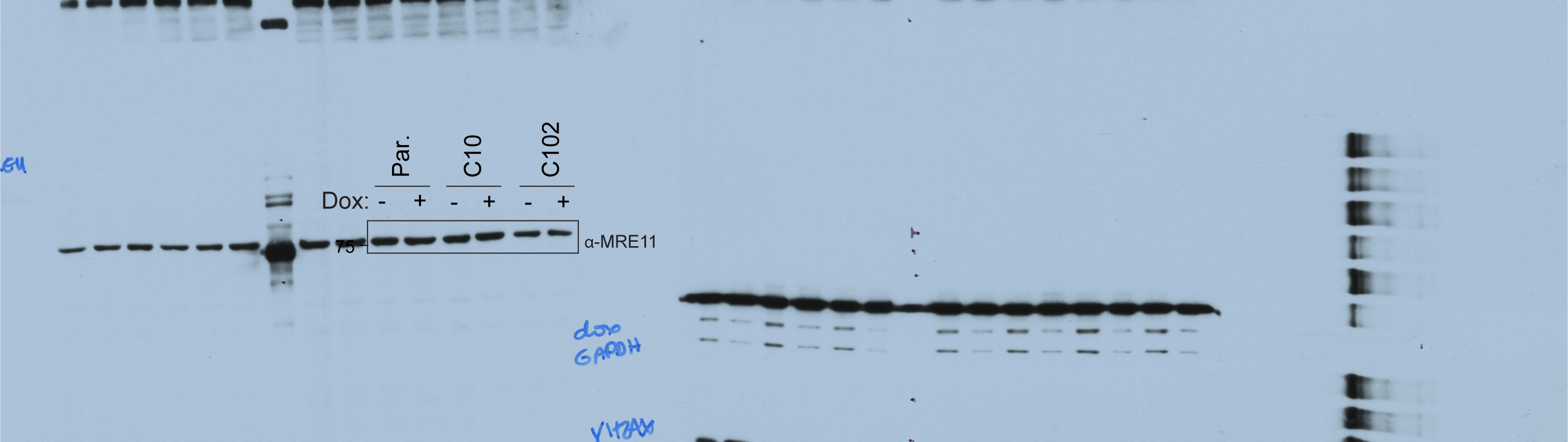

Supplement: Supplementary file 6 — Source Data Fig. 4 [file 44319_2023_35_MOESM6_ESM.zip › Figure 4/4B/image data_anti-MRE11 fig4B.tif]

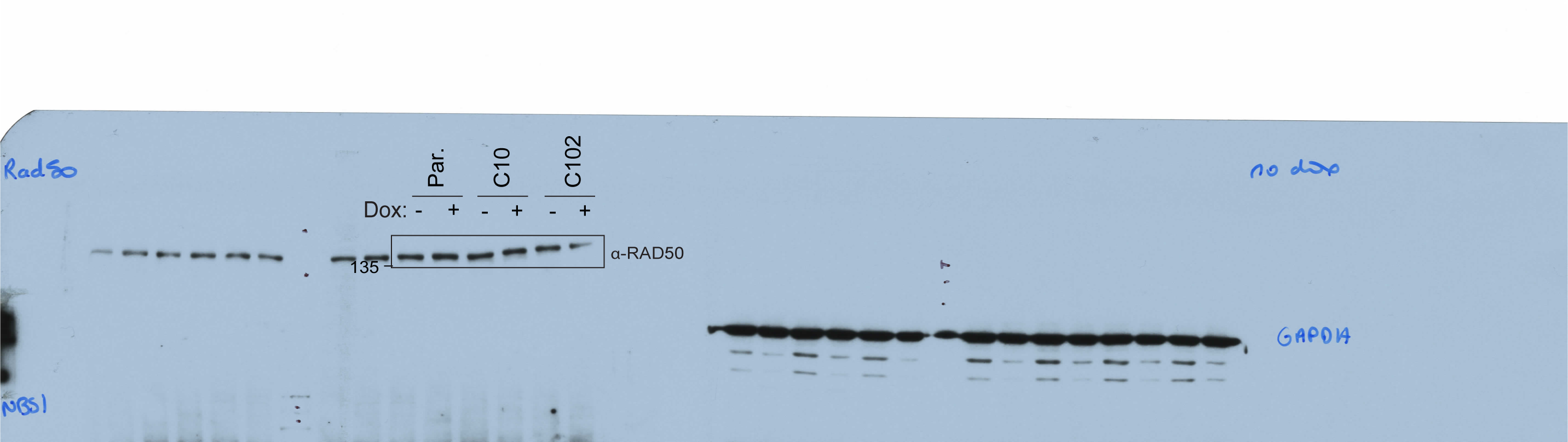

Supplement: Supplementary file 6 — Source Data Fig. 4 [file 44319_2023_35_MOESM6_ESM.zip › Figure 4/4B/image data_anti-RAD50 fig4B.tif]

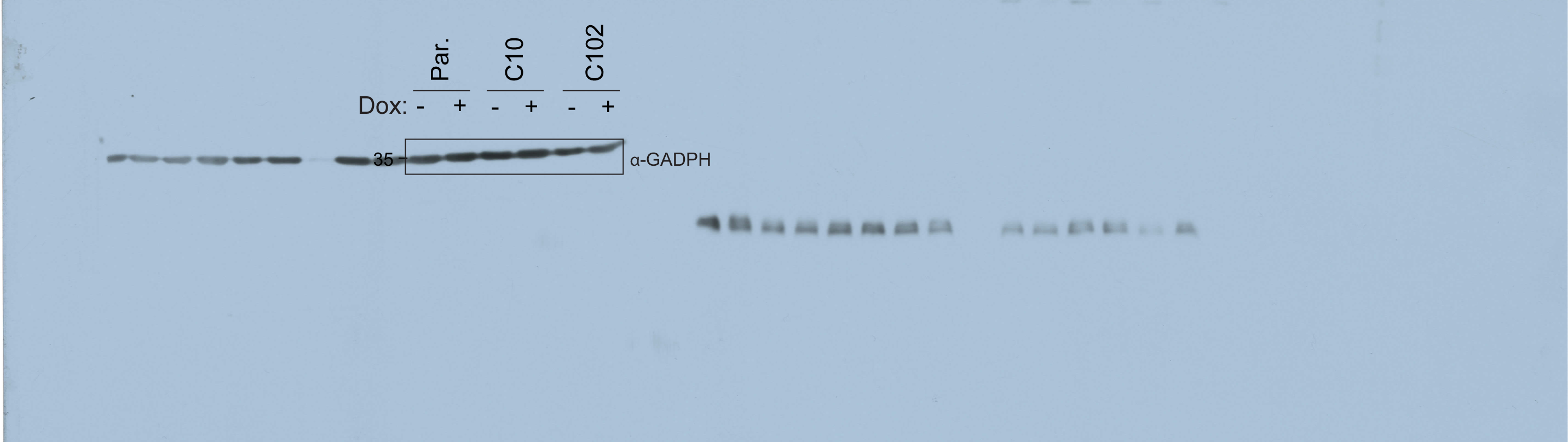

Supplement: Supplementary file 6 — Source Data Fig. 4 [file 44319_2023_35_MOESM6_ESM.zip › Figure 4/4B/image data_anti-GAPDH fig4B.tif]

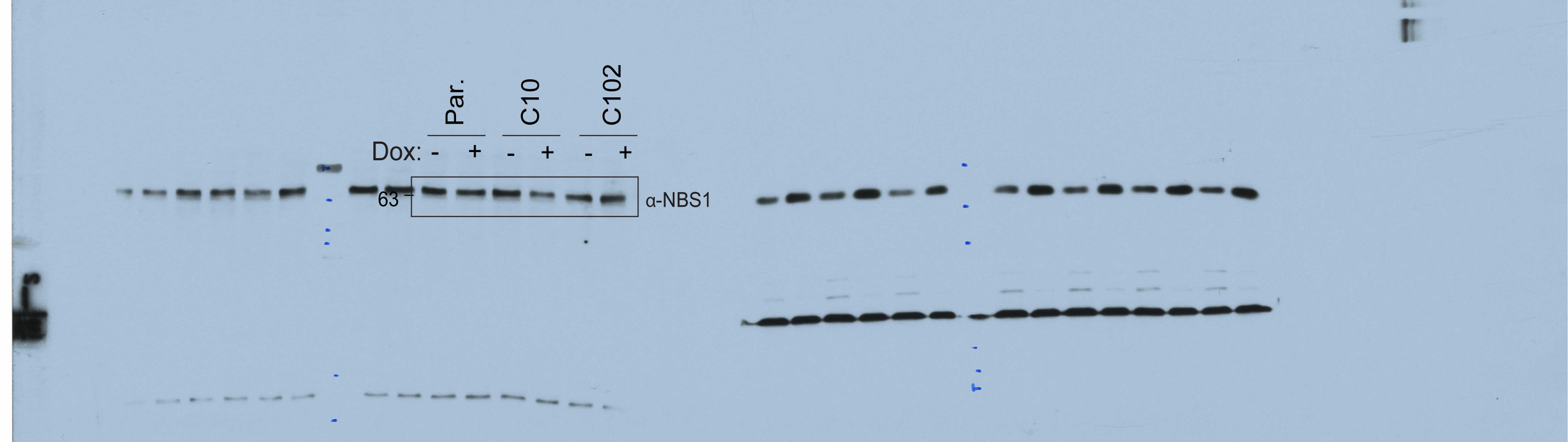

Supplement: Supplementary file 6 — Source Data Fig. 4 [file 44319_2023_35_MOESM6_ESM.zip › Figure 4/4B/image data_anti-NBS1 fig4B.tif]

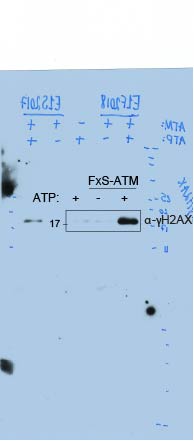

Supplement: Supplementary file 9 — Source Data Fig. 7 [file 44319_2023_35_MOESM9_ESM.zip › Figure 7/7E/image data_anti-yH2AX fig7E.jpg]

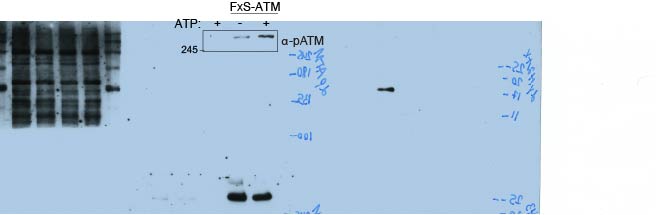

Supplement: Supplementary file 9 — Source Data Fig. 7 [file 44319_2023_35_MOESM9_ESM.zip › Figure 7/7E/image data_anti-pATM fig7E.jpg]

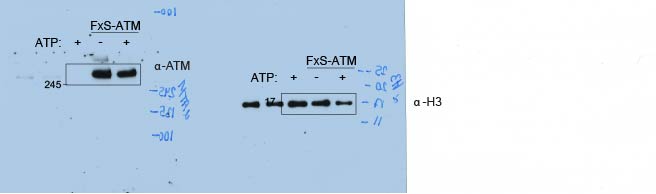

Supplement: Supplementary file 9 — Source Data Fig. 7 [file 44319_2023_35_MOESM9_ESM.zip › Figure 7/7E/image data_anti-ATM and anti-H3 fig7E.jpg]

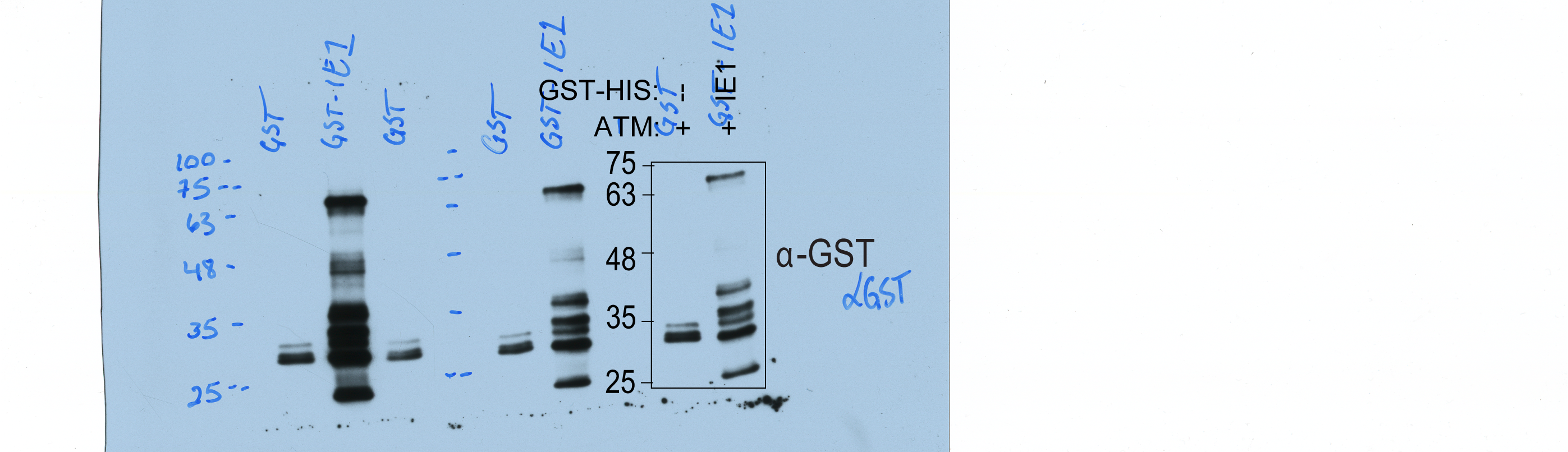

Supplement: Supplementary file 9 — Source Data Fig. 7 [file 44319_2023_35_MOESM9_ESM.zip › Figure 7/7F/image data_anti-GST fig7F.tif]

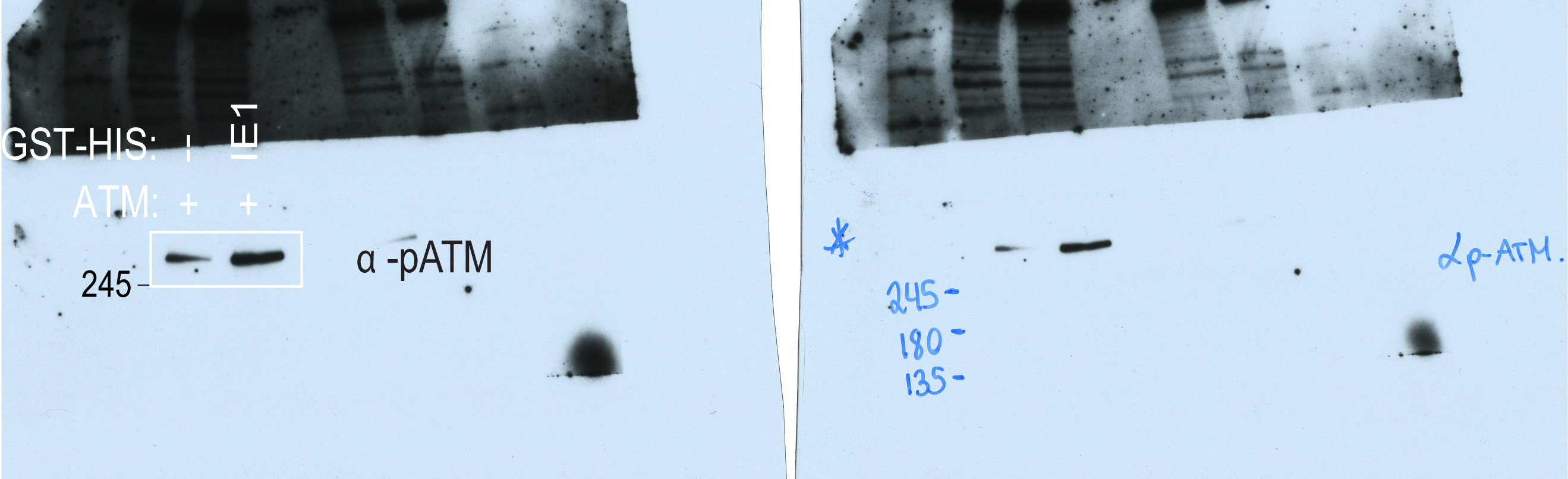

Supplement: Supplementary file 9 — Source Data Fig. 7 [file 44319_2023_35_MOESM9_ESM.zip › Figure 7/7F/image data_anti-pATM Fig7F.tif]

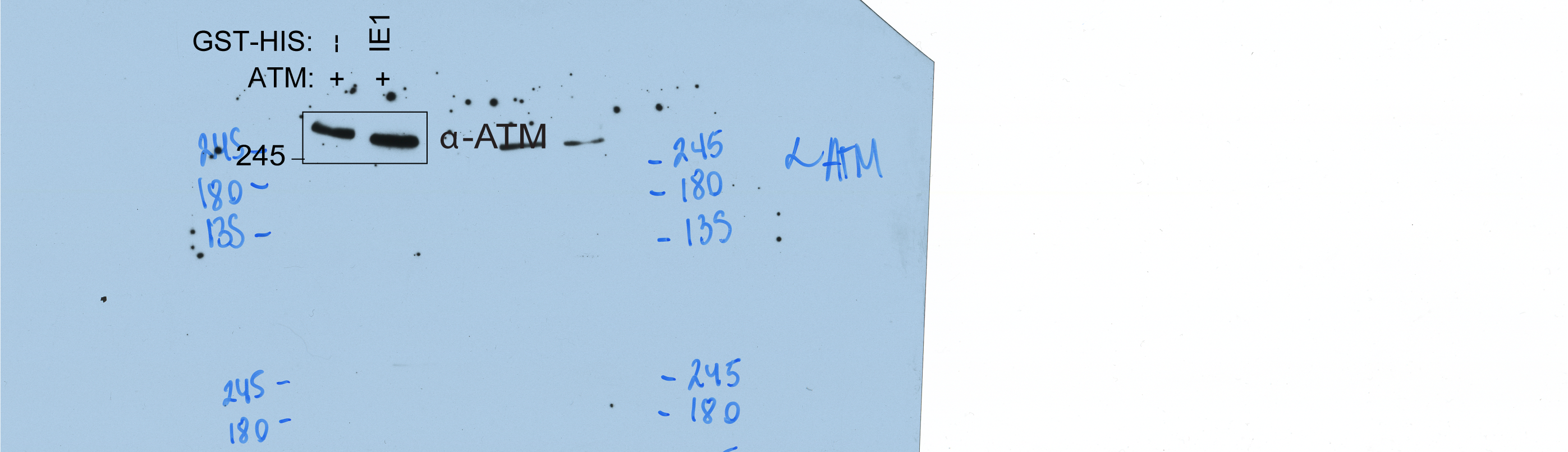

Supplement: Supplementary file 9 — Source Data Fig. 7 [file 44319_2023_35_MOESM9_ESM.zip › Figure 7/7F/image data_anti-ATM fig7F.tif]

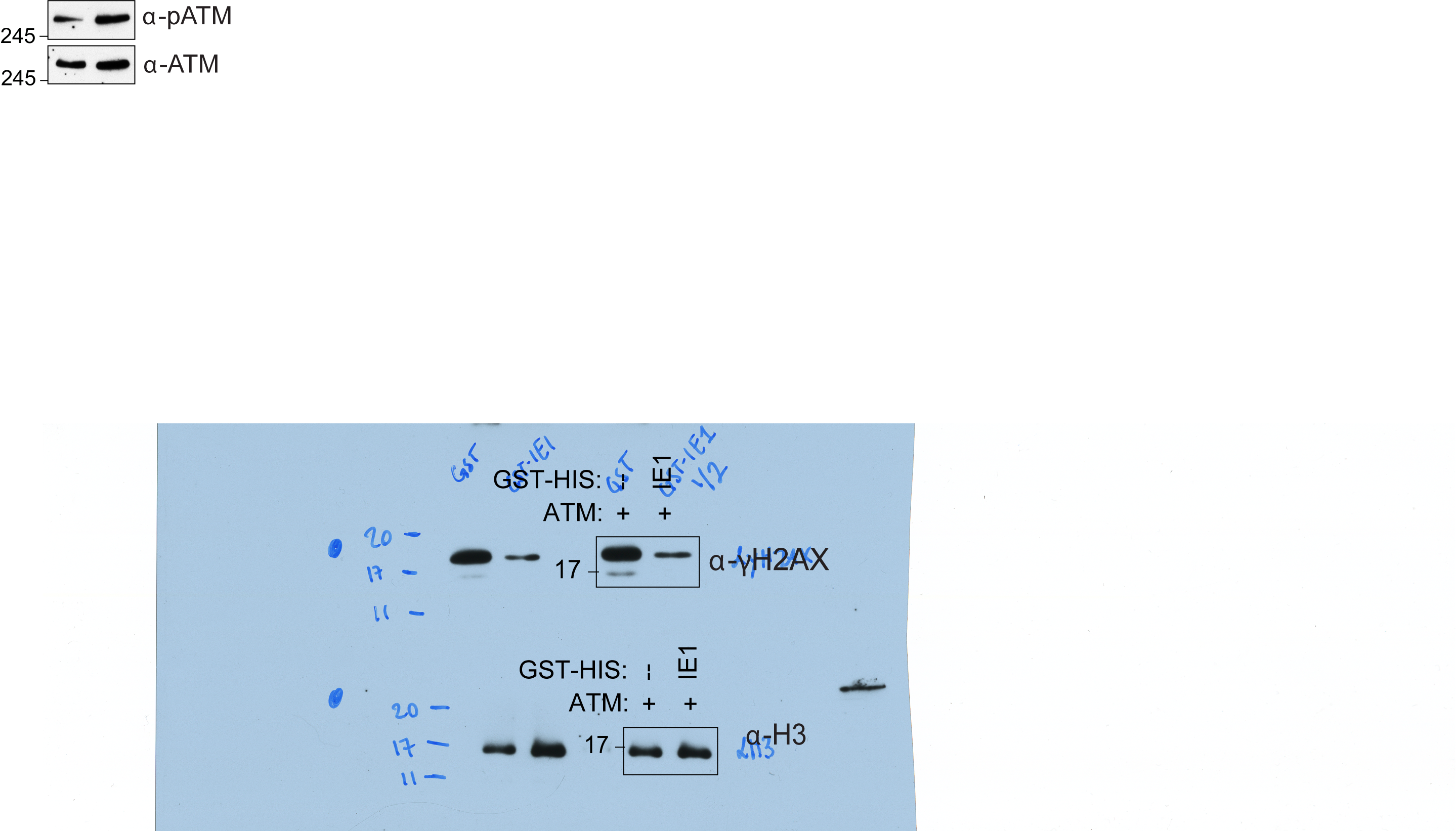

Supplement: Supplementary file 9 — Source Data Fig. 7 [file 44319_2023_35_MOESM9_ESM.zip › Figure 7/7F/image data_anti-H3 and anti-gH2AX fig7F.tif]
